# Hedgehog Signaling Regulates Hypoxia-Associated Metabolic Adaptation in Myeloid Leukemia In Vitro Cell Models

**DOI:** 10.3390/ijms27146324

**Published:** 2026-07-16

**Authors:** Irene Filippi, Sara Monaci, Carlo Aldinucci, Alessandro Falsini, Massimo Bardotti, Federica Coppola, Antonella Naldini, Fabio Carraro

**Affiliations:** 1 Department of Molecular and Developmental Medicine, University of Siena, 53100 Siena, Italy; sara.monaci2@unisi.it (S.M.); carlo.aldinucci@unisi.it (C.A.); alessandro.falsini2@unisi.it (A.F.); massimo.bardotti@student.unisi.it (M.B.); federica.coppola2@unisi.it (F.C.); antonella.naldini@unisi.it (A.N.); 2 Department of Medical Biotechnologies, University of Siena, 53100 Siena, Italy; fabio.carraro@unisi.it

**Keywords:** hypoxia, metabolism, hedgehog, myeloid leukemia cells, AMP-activated protein kinase

## Abstract

Hedgehog (Hh) signaling regulates cell survival and microenvironmental responses in various cancers, but its role in metabolic adaptation to the hypoxic bone marrow microenvironment in myeloid malignancies remains insufficiently defined. K562, KU812, and U937 cells were cultured under normoxic or hypoxic conditions and treated with the Smoothened (Smo) antagonist Cyclopamine or the agonist SAG (Smoothened Agonist). Cell proliferation, apoptosis- and autophagy-related markers, glycolysis-associated proteins, and metabolic parameters were assessed by viability assays, Western blotting, immunofluorescence, and biochemical assays. Selected findings were further evaluated in CRISPR/Cas9-mediated Smo knockout cells. Smo inhibition reduced cell proliferation, increased PARP cleavage, and decreased BNIP3 expression under both oxygen conditions. It also downregulated glucose transporter 1 (GLUT1), hexokinase 2 (HK2), lactate dehydrogenase (LDH), monocarboxylate transporter 1 (MCT1), carbonic anhydrases IX and XII (CAIX and CAXII), and was associated with reduced glucose consumption, lactate production, and ATP levels. These changes were associated with modulation of the AMPK–mTOR axis and with reduced phosphorylation of mTOR downstream effectors. These findings support a role for Hh signaling in hypoxia-driven metabolic adaptation in myeloid malignancy cell models and suggest a functional link between Smo activity, glycolytic remodeling, and AMPK-mTOR-related signaling.

## 1. Introduction

The Hedgehog (Hh) signaling pathway is essential for various developmental processes and organogenesis. As a crucial regulator of cellular growth and differentiation, mutations in its components can lead to aberrant activation, significantly increasing susceptibility to neoplastic transformation.

Consequently, a broad spectrum of small-molecule inhibitors targeting this pathway has been developed for the targeted treatment of various oncological diseases. In the 1950s, studies on congenital defects in lambs led to the discovery of Cyclopamine, a plant-derived steroidal alkaloid with both teratogenic [1] and antitumor properties [2] due to its ability to selectively inhibit cellular responses mediated by Hh signaling through direct binding to the transmembrane receptor Smoothened (Smo).

Preclinical evidence has confirmed that Cyclopamine suppresses the growth of human erythroleukemic cell lines and acute lymphoblastic B-lineage cell lines in vitro [3]. Others reported that Cyclopamine suppressed the growth of some leukemia and lymphoma cells, including acute myeloid leukemia (AML), T-acute lymphoid leukemia (ALL) and B-lymphoma cells, through the induction of apoptosis [4].

In the human body, the oxygen gradient is not uniform across all tissues; it varies from one tissue to another. In bone marrow, these levels are significantly lower than those found in other peripheral organs. The bone marrow microenvironment is characterized by oxygen concentrations ranging from 1% to 7% [5]. Hematopoiesis originates from hematopoietic stem cells (HSCs), which reside in these poorly vascularized areas, developing in a predominantly hypoxic microenvironment. This adaptation is transcriptionally orchestrated by Hypoxia-Inducible Factor 1-alpha (HIF-1α), which reprograms HSC metabolism toward anaerobic glycolysis at the expense of mitochondrial oxidative phosphorylation [5,6,7,8,9].

Although it has been demonstrated that chronic myeloid leukemia (CML) cells retain the ability to survive under hypoxic conditions, the metabolic mechanisms underlying this niche adaptation remain partially undefined.

We previously identified an interaction between the Hh signaling pathway and carbonic anhydrases (CAs), particularly in relation to the migration of melanoma and breast cancer cells [10,11,12,13,14]. Additional studies have shown that CA isoforms IX and XII are essential for the survival of leukemic blasts, enabling them to buffer microenvironmental acidosis, evade apoptosis, and sustain proliferation in severely oxygen-deprived environments [15]. Based on these premises, we hypothesized a potential axis of convergence between Hh signaling and hypoxic metabolic adaptation in myeloproliferative disorders.

Indeed, the relationship between different key metabolic enzymes and hematological malignancies is well known, but the connection with Hh signaling remains largely unexplored, especially under hypoxia.

For instance, overexpression of glucose transporter 1 (GLUT1) has been observed in BCR-ABL-expressing leukemic stem cells compared to healthy counterparts [16], while hexokinase 2 (HK2) has proven essential for the growth of diffuse large B-cell lymphomas under hypoxia [17]. Elevated lactate dehydrogenase (LDH) levels are frequently observed in various human cancers compared to normal tissues, and increased LDH expression has been associated with poor prognosis and drug resistance [18,19]. All these protein-level changes are reflected in functional modifications in glucose consumption, as well as ATP and lactate production. Consistent with the Warburg effect, neoplastic cells adopt a highly glycolytic metabolism, actively secreting lactate into the extracellular space through monocarboxylate transporter 1 (MCT1)-mediated efflux [20,21].

In the signal transduction landscape of hematological malignancies, therapeutic interest in AMP-activated protein kinase (AMPK) is constantly expanding [22]. However, the broad spectrum of AMPK’s molecular targets complicates the interpretation of its phenotypic effects. It is well established that the phosphatidylinositol 3-kinase (PI3K)/Akt/mammalian target of rapamycin (mTOR) axis represents a fundamental hub for anabolic signals in myeloid leukemia and that, under conditions of energy deprivation, mTOR activity is subjected to strict inhibitory control by AMPK. Nevertheless, the potential role of AMPK in mediating the inhibitory effects of Cyclopamine during hypoxic adaptation, as well as the broader crosstalk between Hh and mTORC1 pathways, has never been fully investigated in this context [23,24].

In this study, supported by in vitro models, we demonstrate an interaction between Hh signaling and the metabolic adaptations to a hypoxic microenvironment in myeloid lineages. In particular, inhibiting the Hh pathway, both pharmacologically with Cyclopamine and by CRISPR/Cas9-mediated silencing of Smo, interferes with their metabolic shift toward anaerobic glycolysis. Studying the surrounding microenvironment and targeting hypoxia adaptation mechanisms is of considerable interest, as hypoxia plays a key role in promoting resistance to anticancer therapies.

To this end, we employed three human myeloid leukemia cell lines. Two of them, K562 and KU812, are well-established models of chronic myeloid leukemia (CML), both harboring the Philadelphia chromosome and the BCR-ABL1 fusion gene, although they differ in lineage and phenotypic characteristics. In contrast, the U937 cell line is Philadelphia chromosome-negative and lacks the BCR-ABL1 fusion gene. The presence of BCR-ABL1 in K562 and KU812 confers constitutive tyrosine kinase activity, driving uncontrolled proliferation, resistance to apoptosis, and oncogenic transformation, while also rendering these cells sensitive to tyrosine kinase inhibitors (TKIs), although resistance can arise in clinical settings.

The analysis of alternative pathways, such as Hedgehog signaling under hypoxia, offers an invaluable perspective on the metabolic regulation of CML, identifying paradigms of biochemical vulnerability that may be shared across different myeloid pathologies, independent of molecular reliance on BCR-ABL1.

## 2. Results

### 2.1. The Hh Pathway Is Involved in K562 Cell Survival

CML is a myeloproliferative neoplasm characterized by dysregulated proliferation of hematopoietic stem cells [25]. Since Hh signaling has been implicated in cell proliferation and survival [26], we sought to investigate its potential role in modulating cell survival and proliferation in CML cells, under both normoxic and hypoxic (1% O_2_) conditions. Indeed, hematopoietic stem cells originate and proliferate in the bone marrow, an environment with low oxygen levels ranging from 4% to nearly zero [27].

**Figure 1:**
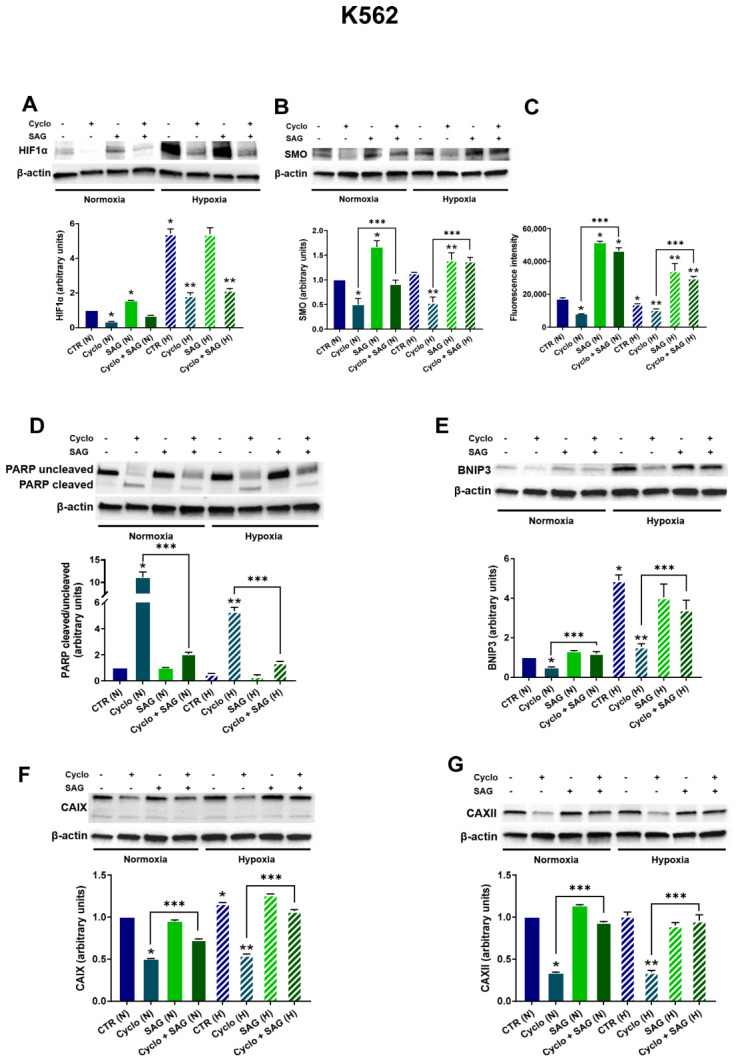
Cyclopamine impairs K562 cell survival under normoxic and hypoxic conditions. K562 cells were treated with Cyclopamine or SAG under normoxic or hypoxic conditions for 48 h. Protein expression levels of HIF-1α (**A**), SMO (**B**), uncleaved and cleaved PARP (**D**), BNIP3 (**E**), CAIX (**F**), and CAXII (**G**) were analyzed by Western blotting. β-actin served as the loading control. The same β-actin loading control was used for target proteins detected on membranes that were stripped and reprobed with different antibodies. A representative blot is shown from three independent experiments. (**C**) Cell viability was assessed using the CyQuant assay. Data represent means ± SEM (*n* = 3; * *p* ≤ 0.05 indicates statistical significance compared to normoxic control, ** *p* ≤ 0.05 vs. hypoxic control, *** *p* ≤ 0.05 vs. Cyclopamine treatment). CTR = control, Cyclo = Cyclopamine.

To verify the hypoxic conditions and investigate the relationship between hypoxia and Hh signaling we studied the expression of Hypoxia-Inducible Factor (HIF)-1α, the main regulator of adaptive responses to a hypoxic microenvironment. The Western blot in Figure 1A confirmed the elevated expression of HIF-1α under hypoxia at 48 h. It also showed a significant inhibition with Cyclopamine (a Smo antagonist) incubation.

To assess the involvement of the Hh pathway, K562 cells were treated with either Cyclopamine or SAG (a Smo agonist) for 48 h under both normoxia and hypoxia, followed by analysis of Smo protein levels. As shown in Figure 1B, Cyclopamine treatment significantly reduced Smo protein levels, whereas SAG increased them. Notably, co-treatment with both Cyclopamine and SAG restored Smo levels, indicating functional antagonism. Furthermore, Cyclopamine treatment suppressed glioma-associated oncogene (GLI) expression at both 48 and 72 h under normoxic and hypoxic conditions, confirming inhibition of canonical Hh signaling (Supplementary Figure S1).

Next, we evaluated the effects of these treatments on cell proliferation using the CyQuant assay. As depicted in Figure 1C, Cyclopamine significantly reduced K562 cell proliferation, whereas SAG promoted proliferation in both normoxic and hypoxic conditions. Importantly, co-administration of SAG reversed the anti-proliferative effects of Cyclopamine, highlighting the opposing roles of these compounds in modulating Hh signaling. Of note, hypoxia itself reduced cell proliferation compared to normoxic conditions. All experiments were performed at the 48-h time point, at which the divergence between normoxic and hypoxic proliferation becomes evident: cells cultured in hypoxia exhibit a proliferation arrest, while those in normoxia continue to increase in number.

To elucidate the mechanisms underlying Cyclopamine-mediated suppression of K562 cell proliferation, we examined Poly(ADP-ribose) polymerase (PARP) cleavage, a hallmark of apoptosis. As shown in Figure 1D, in both oxygen conditions, Cyclopamine treatment significantly increased the levels of cleaved PARP, suggesting a potential trend of apoptotic cell death.

Additionally, we investigated the expression of Bcl-2/adenovirus E1B 19 kDa-interacting protein 3 (BNIP3), a critical regulator of autophagy. As demonstrated in Figure 1E, Cyclopamine treatment markedly decreased BNIP3 levels, while neither SAG alone nor the combination of Cyclopamine and SAG had any significant impact, in conditions of both normoxia and hypoxia. This points to a potential involvement of autophagy-related pathways, although further functional assays would be required to clarify its specific impact on autophagic flux.

Previous studies from our group have established correlations between CAs and Hh pathway activity in breast cancer [13,14] and melanoma [10,11,12]. Based on these findings, we evaluated the expression of CAIX and CAXII in our CML model. As shown in Figure 1F,G, Cyclopamine treatment significantly downregulated both CAIX and CAXII under the two oxygen conditions tested. In contrast, neither SAG nor combined SAG and Cyclopamine treatments elicited significant changes in their expression.

### 2.2. Alterations in Hh Signaling Induce Metabolic Changes Under Normoxic and Hypoxic Conditions

To survive and adapt to their microenvironment, cancer cells undergo metabolic reprogramming, often shifting to a glycolysis-dominant metabolic profile [20]. Emerging evidence highlights the interplay between Hh signaling and cellular metabolism [28,29,30,31,32]. Based on these findings, we sought to explore the potential relationship between metabolic adaptations and Hh signaling in K562 cells.

**Figure 2:**
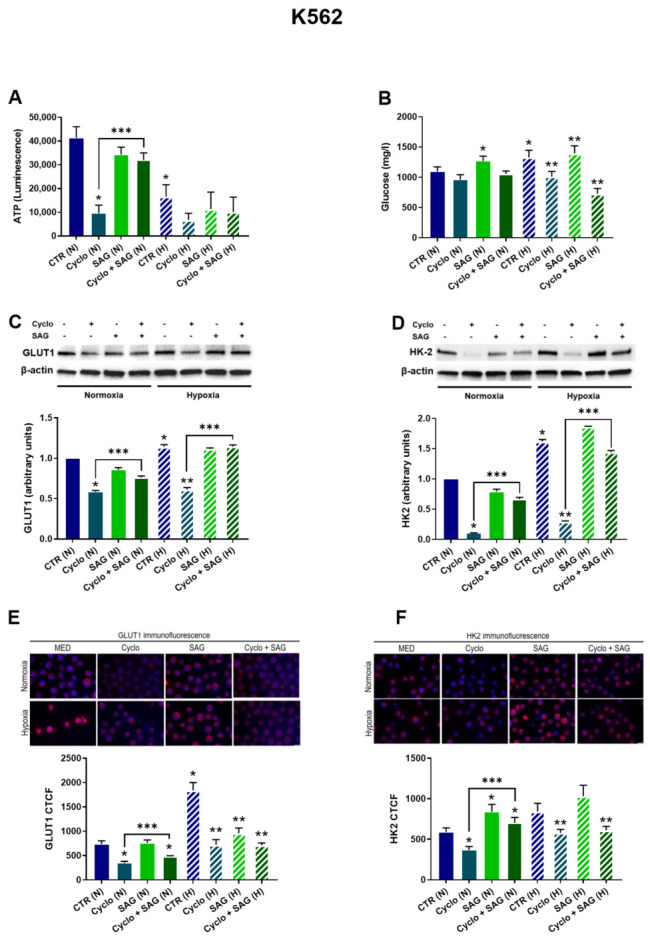
Modulation of Hh signaling induces metabolic changes in K562 cells under normoxic and hypoxic conditions. K562 cells were treated with Cyclopamine or SAG under normoxic or hypoxic conditions for 48 h. ATP (**A**) and glucose (**B**) levels were measured using ATP-Lite and Amplex Red glucose detection assays. Protein expression levels of GLUT1 (**C**) and HK2 (**D**) were analyzed by Western blotting. β-actin served as the loading control. A representative blot is shown from three independent experiments. Immunofluorescence images of GLUT1 (**E**) and HK2 (**F**) were acquired using an Olympus IX81 microscope (60× magnification, scale bar: 10 µm). Data represent means ± SEM (*n* = 3; * *p* ≤ 0.05 vs. normoxic control, ** *p* ≤ 0.05 vs. hypoxic control, *** *p* ≤ 0.05 vs. Cyclopamine treatment). CTR = control, Cyclo = Cyclopamine, N = normoxia, H = hypoxia.

First, ATP levels were measured after 48 h of treatment. Cyclopamine reduced ATP levels in K562 cells under both normoxic and hypoxic conditions (Figure 2A). In contrast, neither SAG alone nor the combination of SAG and Cyclopamine elicited significant changes. As anticipated, ATP levels were lower in hypoxia than in normoxia, consistent with the shift toward anaerobic metabolism.

Since glucose serves as the primary energy substrate for cellular metabolism, we assessed glucose consumption. Figure 2B illustrated that glucose utilization was decreased in the presence of Cyclopamine and this reduction became significant in hypoxic conditions. Conversely, SAG treatment increased glucose consumption. GLUT1, a key regulator of glycolysis, was analyzed after 48 h of treatment. Cyclopamine significantly reduced GLUT1 protein expression in both normoxic and hypoxic conditions (Figure 2C). In contrast, SAG treatment, either alone or in combination with Cyclopamine, did not affect GLUT1 levels. Immunofluorescence analysis (Figure 2E) corroborated these findings, showing marked downregulation of GLUT1 in Cyclopamine-treated cells under both normoxia and hypoxia. Notably, GLUT1 expression was upregulated in hypoxia relative to normoxia, consistent with its regulation by hypoxia-responsive elements (HREs) [33].

HK2, another critical enzyme in glucose metabolism, displayed a similar trend to GLUT1. Western blot (Figure 2D) and immunofluorescence analysis (Figure 2F) revealed that Cyclopamine significantly suppressed HK2 expression under both normoxic and hypoxic conditions at 48 h.

Collectively, these findings suggest a potential link between Hh signaling and metabolic adaptations to the hypoxic microenvironment in K562 cells.

### 2.3. Modulation of Hh Signaling Alters Lactate Metabolism Under Normoxic and Hypoxic Conditions

Lactate, the end product of both anaerobic and aerobic glycolysis, is generated from pyruvate by LDH. Once considered a mere byproduct of glycolysis, emerging evidence suggests that lactate serves as a critical energy source [34,35].

**Figure 3:**
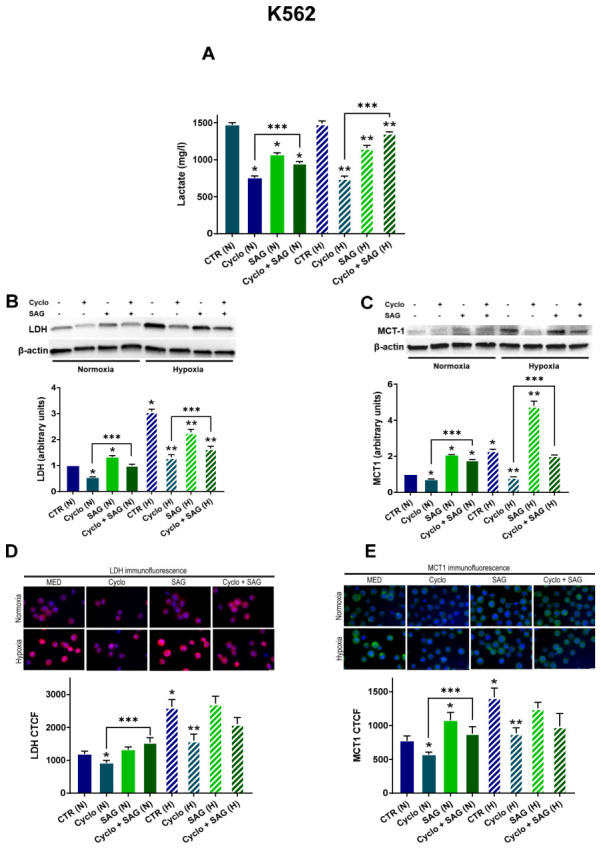
Cyclopamine modulates lactate metabolism in K562 cells under normoxic and hypoxic conditions. K562 cells were treated with Cyclopamine or SAG under normoxic or hypoxic conditions for 48 h. Lactate production (**A**) was measured using the Amplex Red lactate detection assay. Protein expression levels of LDH (**B**) and MCT1 (**C**) were analyzed by Western blotting. β-actin served as the loading control. A representative blot from three independent experiments is shown. Immunofluorescence images of LDH (**D**) and MCT1 (**E**) were acquired using an Olympus IX81 microscope (60× magnification, scale bar: 10 µm). Data represent means ± SEM (*n* = 3; * *p* ≤ 0.05 vs. normoxic control, ** *p* ≤ 0.05 vs. hypoxic control, *** *p* ≤ 0.05 vs. Cyclopamine treatment). CTR = control, Cyclo = Cyclopamine, N = normoxia, H = hypoxia.

To assess the impact of Cyclopamine on lactate production, we measured lactate efflux after 48 h. As shown in Figure 3A, Cyclopamine significantly inhibited lactate efflux under both normoxic and hypoxic conditions, consistent with its suppression of Smo signaling. Western blot (Figure 3B) and immunofluorescence analysis (Figure 3D) were performed to evaluate LDH expression in K562 cells after 48 h of treatment. Compared to normoxic controls, hypoxia significantly increased LDH protein levels, consistent with its regulation by HIF-1α. However, Cyclopamine significantly reduced LDH expression under both normoxic and hypoxic conditions, whereas SAG alone or combined with Cyclopamine induced opposite or less evident effects on LDH levels.

MCT-1, a glycolysis-associated protein involved in lactate transport, exhibited expression patterns consistent with those of LDH. Cyclopamine significantly inhibited MCT-1 expression under both normoxic and hypoxic conditions relative to respective controls, whereas SAG treatment increased MCT-1 levels (Figure 3C). Immunofluorescence analysis (Figure 3E) confirmed these findings, aligning with the Western blot results. Taken together, these data indicate that Cyclopamine suppresses glucose consumption and reduces lactate and ATP production in K562 cells.

### 2.4. Modulation of Hh Signaling Under Normoxic and Hypoxic Conditions Impairs Cell Survival and Induces Metabolic Reprogramming in KU812 Cells

**Figure 4:**
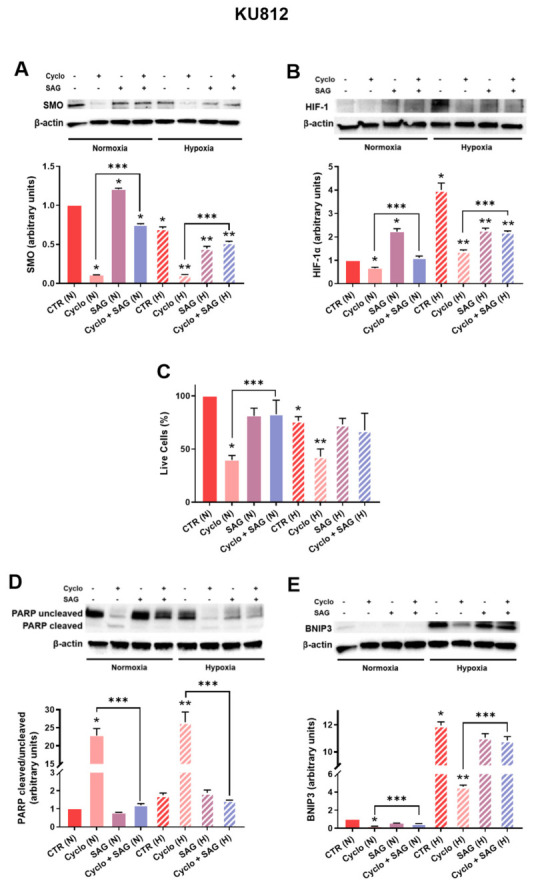
Modulation of Hh signaling impairs cell survival in KU812 cells under normoxic and hypoxic conditions. KU812 cells were analyzed after Cyclopamine and SAG treatments under normoxia or hypoxia for 48 h. Protein expression levels of SMO (**A**), HIF-1α (**B**), PARP uncleaved and cleaved (**D**), and BNIP3 (**E**), were determined by Western blotting. β-actin was used as loading control. The same β-actin loading control was used for target proteins detected on membranes that were stripped and reprobed with different antibodies. Blots are representative of three independent experiments. (**C**) Cell viability was determined by trypan blue exclusion. Means ± SEM are presented (*n* = 3; * *p* ≤ 0.05 vs. normoxic control, ** *p* ≤ 0.05 vs. hypoxic control, *** *p* ≤ 0.05 vs. Cyclopamine treatment). CTR = control, Cyclo = Cyclopamine, N = normoxia, H = hypoxia.

To validate the findings observed in K562 cells, we performed analogous experiments using the myeloid cell line KU812. As shown in Figure 4A, Cyclopamine significantly downregulated Smo expression under both normoxic and hypoxic conditions after 48 h, whereas SAG or combined treatment with SAG and Cyclopamine did not elicit similar effects.

Hypoxic conditions were confirmed by the upregulation of HIF-1α protein levels under hypoxia. Consistent with observations in K562 cells, Cyclopamine significantly suppressed HIF-1α expression, especially under hypoxic conditions, whereas SAG alone or in combination with Cyclopamine exerted opposite or only minor effects (Figure 4B).

As depicted in Figure 4C, only Cyclopamine treatment significantly reduced KU812 cell proliferation after 48 h, as assessed by the trypan blue exclusion method. This inhibitory effect was observed under both normoxic and hypoxic conditions.

Consistent with findings in K562 cells, Cyclopamine treatment in KU812 cells was associated with increased cleavage of the pro-apoptotic protein PARP (Figure 4D) and decreased expression of the pro-autophagy protein BNIP3 (Figure 4E), specifically under both normoxic and hypoxic conditions.

**Figure 5:**
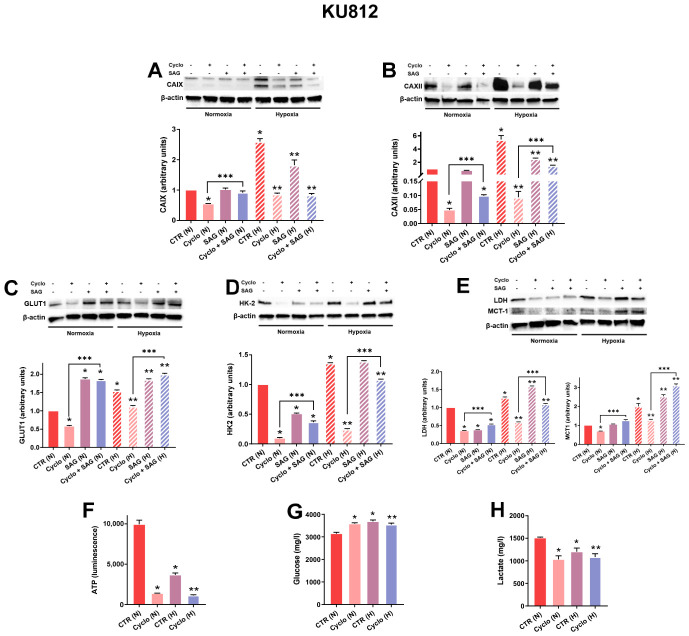
Modulation of Hh signaling impairs cell metabolism in KU812 cells under normoxic and hypoxic conditions. KU812 cells were analyzed after Cyclopamine and SAG treatments under normoxia or hypoxia for 48 h. Protein expression levels of CAIX (**A**), CAXII (**B**), GLUT1 (**C**), HK2 (**D**), LDH and MCT1 (**E**) were determined by Western blotting. β-actin was used as loading control. Blots are representative of three independent experiments. Measurements of ATP (**F**), glucose (**G**) and lactate (**H**) levels were done by using ATP-Lite and Amplex-red glucose or lactate detection assays. Means ± SEM are presented (*n* = 3; * *p* ≤ 0.05 vs. normoxic control, ** *p* ≤ 0.05 vs. hypoxic control, *** *p* ≤ 0.05 vs. Cyclopamine treatment). CTR = control, Cyclo = Cyclopamine, N = normoxia, H = hypoxia.

In addition to impaired cell survival, Cyclopamine treatment reduced the expression of proteins involved in microenvironment acidification, such as CAs. Specifically, Cyclopamine significantly downregulated both CAIX (Figure 5A) and CAXII (Figure 5B) in KU812 cells under normoxic and hypoxic conditions after 48 h.

Given the established link between Hh signaling and glucose/lactate metabolism, we examined the expression of key metabolic proteins in KU812 cells. After 48 h of Cyclopamine treatment, the expression of GLUT1 (Figure 5C) and HK2 (Figure 5D) was significantly reduced under both normoxic and hypoxic conditions. In contrast, neither SAG nor combined treatment with SAG and Cyclopamine elicited similar effects.

Western blot analysis (Figure 5E) revealed that Cyclopamine treatment significantly reduced the expression of both LDH and MCT1 proteins relative to respective controls under normoxic and hypoxic conditions.

These findings confirm that Cyclopamine treatment impairs significantly the expression of key glycolytic proteins in KU812 cells, whereas SAG alone or in combination with Cyclopamine exerts opposite or less pronounced effects.

To determine whether these changes correlate with functional metabolic adaptations, we assessed ATP content, glucose consumption and lactate production. ATP content was measured to evaluate energy status. As shown in Figure 5F, Cyclopamine treatment significantly reduced ATP production under both normoxic and hypoxic conditions. To determine whether Cyclopamine inhibits glucose uptake in this cell model, we performed glucose consumption assays after 48 h. As shown in Figure 5G, Cyclopamine-treated KU812 cells exhibited significantly reduced glucose consumption compared to controls, only under hypoxic conditions where energy status was compromised. The downregulation of glycolytic markers was accompanied by a significant reduction in lactate levels in Cyclopamine-treated cells under both normoxic and hypoxic conditions (Figure 5H).

In conclusion, our findings demonstrate that modulation of the Hh pathway elicits similar metabolic responses in both K562 and KU812 myeloid cell lines.

### 2.5. Potential Pathways Linking Hh Signaling to Metabolic Regulation

**Figure 6:**
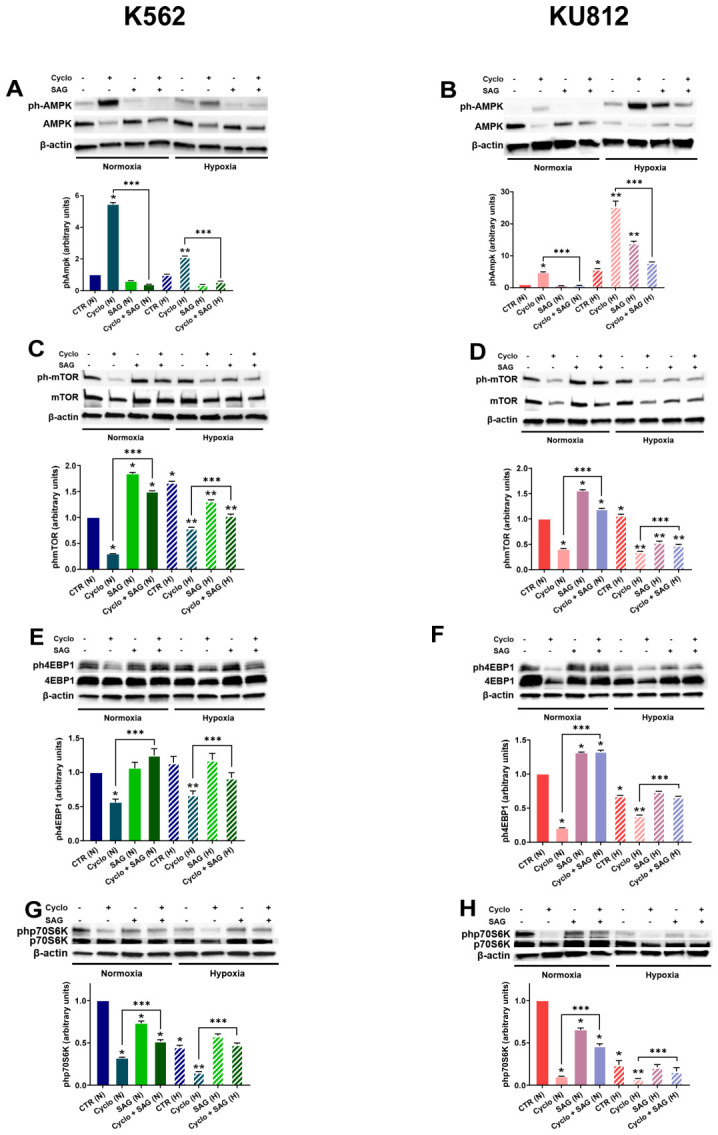
Potential pathways linking Hh signaling to metabolic regulation under normoxic and hypoxic conditions. Cells were treated with Cyclopamine and SAG in normoxic or hypoxic conditions for 48 h. ph-AMPK/AMPK expression was determined by Western blotting in K562 (**A**) and KU812 (**B**) cells. Using Western blot analyses, ph-mTOR/mTOR expression was detected in K562 (**C**) and KU812 (**D**) cells. Protein expression levels of ph4EBP1/4EBP1 in K562 (**E**) and KU812 (**F**) cells were determined by Western blotting. ph-p70S6K/p70S6K expression was analysed by Western blotting in K562 (**G**) and KU812 (**H**) cells. β-actin was used as loading control. The same β-actin loading control was used for target proteins detected on membranes that were stripped and reprobed with different antibodies. Blots are representative of three independent experiments. Means ± SEM are presented (*n* = 3; * *p* ≤ 0.05 vs. normoxic control, ** *p* ≤ 0.05 vs. hypoxic control, *** *p* ≤ 0.05 vs. Cyclopamine treatment). CTR = control, Cyclo = Cyclopamine, N = normoxia, H = hypoxia.

Finally, we explored the pathways potentially underlying the metabolic alterations observed following Cyclopamine treatment. AMPK is a well-established sensor of cellular energy status [36,37] and Smo modulators have been shown to uncouple the Smo-AMPK axis from the canonical Hedgehog signaling pathway [38]. However, the interplay between these pathways in myeloid lineages, particularly under hypoxic conditions, remains poorly understood. In our study, 48-h Cyclopamine treatment significantly increased AMPK phosphorylation while reducing total AMPK expression in both K562 (Figure 6A) and KU812 (Figure 6B) cell lines under normoxic and hypoxic conditions. These effects were not observed with SAG treatment or combined treatment with SAG and Cyclopamine. To elucidate the molecular mechanisms underlying Cyclopamine-mediated inhibition of myeloid cell proliferation, we examined mTOR, a key regulator of cell survival downstream of AMPK. As shown in Figure 6C,D, Cyclopamine significantly reduced mTOR phosphorylation respectively in both K562 and KU812 cells under normoxic and hypoxic conditions.

In line with the observed suppression of mTOR activity, we next assessed two canonical downstream effectors of mTOR signaling, 4EBP1 and p70S6K, which play central roles in translational control. As shown in Figure 6E,F, Cyclopamine significantly reduced 4EBP1 phosphorylation in both K562 and KU812 cells under normoxic and hypoxic conditions. In contrast, neither SAG treatment nor the combined SAG/Cyclopamine treatment produced significant changes, except for significant changes in the opposite direction. A similar pattern was observed for p70S6K phosphorylation (Figure 6G,H). Cyclopamine consistently inhibited php70S6K levels in both cell lines, while the other treatment conditions did not exert comparable effects.

Collectively, these findings suggest that the AMPK-mTOR axis may play a critical role in mediating Hh signaling-driven metabolic adaptations in response to hypoxic microenvironments in myeloid lineages.

### 2.6. Modulation of Hh Signaling Reveals Conserved but Attenuated Metabolic Responses in U937 Cells, in Both Normoxia and Hypoxia

**Figure 7:**
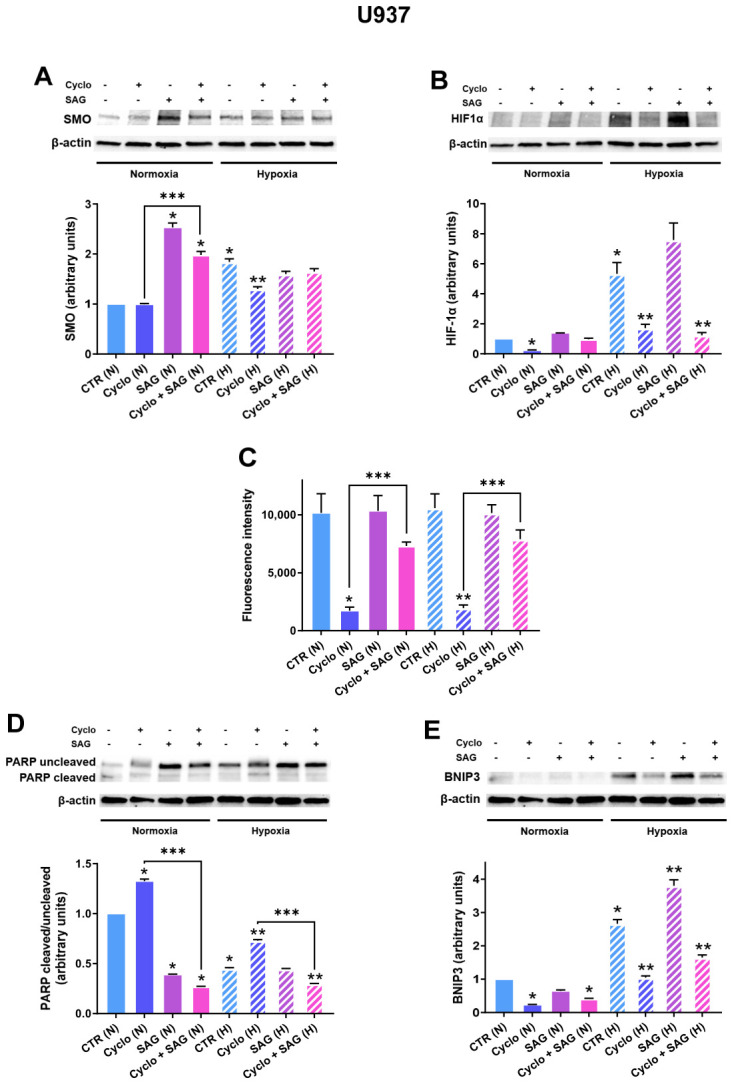
Modulation of Hh signaling reveals conserved survival responses in U937 cells, in both normoxia and hypoxia. U937 cells were analyzed after Cyclopamine and SAG treatments under normoxia or hypoxia for 48 h. Protein expression levels of SMO (**A**), HIF-1α (**B**), PARP uncleaved and cleaved (**D**), and BNIP3 (**E**) were determined by Western blotting. β-actin was used as loading control. The same β-actin loading control was used for target proteins detected on membranes that were stripped and reprobed with different antibodies. Blots are representative of three independent experiments. (**C**) Cell viability was assessed using the CyQuant assay. Means ± SEM are presented (*n* = 3; * *p* ≤ 0.05 vs. normoxic control, ** *p* ≤ 0.05 vs. hypoxic control, *** *p* ≤ 0.05 vs. Cyclopamine treatment). CTR = control, Cyclo = Cyclopamine, N = normoxia, H = hypoxia.

To determine whether the effects observed in BCR-ABL+ CML cell lines (K562 and KU812) were specific to leukemic transformation or represented a broader myeloid response, we investigated the impact of Hedgehog pathway modulation in U937 cells, a non–BCR-ABL+, non-CML myeloid lineage used here as a control. As shown in Figure 7A, Cyclopamine efficiently reduced Smo expression under both normoxic and hypoxic conditions after 48 h, whereas SAG enhanced its expression, confirming that U937 cells remain responsive to Smo inhibition.

Hypoxic conditions were validated by the expected stabilization of HIF-1α, and Cyclopamine markedly reduced HIF-1α expression, particularly under hypoxia (Figure 7B).

Consistent with the behavior observed in CML lines, only Cyclopamine treatment significantly impaired U937 cell proliferation after 48 h (Figure 7C), indicating that Hh pathway inhibition affects cell viability independently of the BCR-ABL oncogenic background. In association, Cyclopamine exposure resulted in increased PARP cleavage (Figure 7D) and reduced BNIP3 protein levels (Figure 7E) under normoxia and hypoxia.

**Figure 8:**
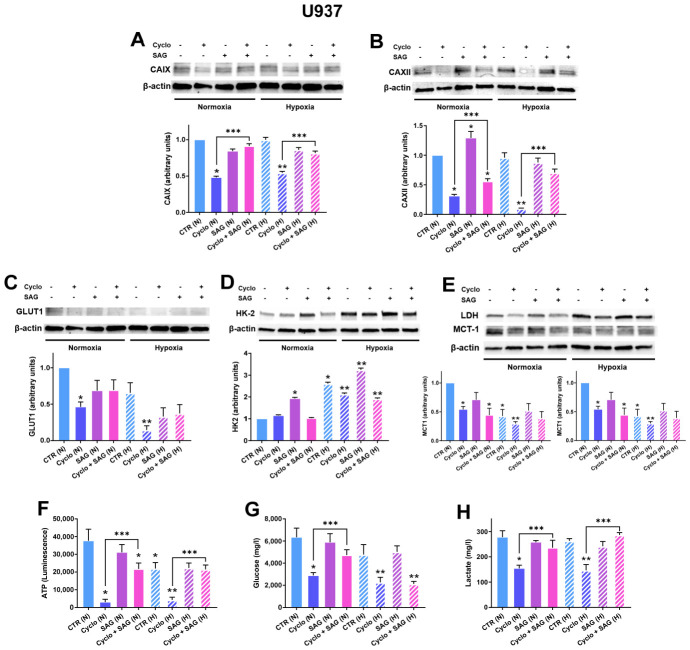
Modulation of Hh signaling reveals conserved but attenuated metabolic responses in U937 cells, in both normoxia and hypoxia. U937 cells were analyzed after Cyclopamine and SAG treatments under normoxia or hypoxia for 48 h. Protein expression levels of CAIX (**A**), CAXII (**B**), GLUT1 (**C**), HK2 (**D**), LDH and MCT1 (**E**) were determined by Western blotting. β-actin was used as loading control. The same β-actin loading control was used for target proteins detected on membranes that were stripped and reprobed with different antibodies. Blots are representative of three independent experiments. Measurements of ATP (**F**), glucose (**G**) and lactate (**H**) levels were done by using ATP-Lite and Amplex-red glucose or lactate detection assays. Means ± SEM are presented (*n* = 3; * *p* ≤ 0.05 vs. normoxic control, ** *p* ≤ 0.05 vs. hypoxic control, *** *p* ≤ 0.05 vs. Cyclopamine treatment). CTR = control, Cyclo = Cyclopamine, N = normoxia, H = hypoxia.

Additionally, Cyclopamine reduced the expression of microenvironment-acidifying carbonic anhydrases CAIX (Figure 8A) and CAXII (Figure 8B), supporting a consistent role for Hh signaling in regulating pH-modulating enzymes across myeloid cell types.

To further examine metabolic reprogramming, we evaluated glycolytic protein expression. Cyclopamine significantly decreased protein levels of GLUT1 (Figure 8C) and HK2 (Figure 8D) after 48 h of hypoxia exposure, whereas SAG alone or in combination with Cyclopamine had no relevant effect. Similarly, expression of LDH and monocarboxylate transporter MCT1 was markedly reduced following Cyclopamine treatment (Figure 8E), confirming that Hh inhibition suppresses key glycolytic components even in the absence of BCR-ABL oncogenic signaling.

To determine whether these molecular changes translated into functional metabolic alterations, ATP content, glucose consumption, and lactate production were quantified. As shown in Figure 8F, Cyclopamine significantly decreased ATP levels under both normoxic and hypoxic conditions. Glucose consumption (Figure 8G) and lactate production were similarly reduced in Cyclopamine-treated cells across oxygen conditions (Figure 8H), in line with the downregulation of glycolytic markers.

Collectively, these findings indicate that although U937 cells lack BCR-ABL and do not represent a CML model, they exhibit comparable metabolic and survival responses to Hh pathway inhibition, reinforcing the concept that the Hedgehog axis rules fundamental metabolic adaptations in myeloid lineages independently of leukemic transformation.

### 2.7. AMPK–mTOR Signaling in U937 Cells Reveals a Cell-Type-Specific Response to Hh Inhibition

**Figure 9:**
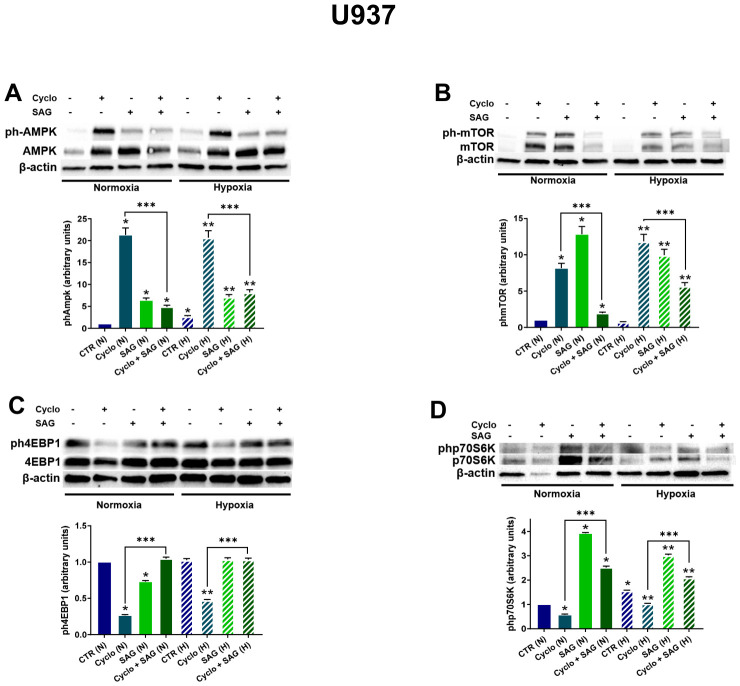
AMPK–mTOR signaling in U937 cells reveals a cell-type-specific response to Hh inhibition. U937 cells were treated with Cyclopamine and SAG in normoxic or hypoxic conditions for 48 h. Protein expression levels of ph-AMPK/AMPK (**A**), ph-mTOR/mTOR (**B**), ph4EBP1/4EBP1 (**C**), and ph-p70S6K/p70S6K (**D**) were determined by Western blotting. β-actin was used as loading control. Blots are representative of three independent experiments. Means ± SEM are presented (*n* = 3; * *p* ≤ 0.05 vs. normoxic control, ** *p* ≤ 0.05 vs. hypoxic control, *** *p* ≤ 0.05 vs. Cyclopamine treatment). CTR = control, Cyclo = Cyclopamine, N = normoxia, H = hypoxia.

To determine whether the AMPK–mTOR axis responds uniformly to Hedgehog pathway modulation across different myeloid contexts, we examined U937 cells as a control. As shown in Figure 9A, 48-h Cyclopamine treatment increased AMPK phosphorylation under both normoxic and hypoxic conditions, emulating the response observed in K562 and KU812 cells. This confirms that AMPK activation represents a conserved outcome of Smo inhibition.

However, the behavior of mTOR differed markedly from that observed in the two CML models. In contrast to K562 and KU812, where Cyclopamine strongly reduced mTOR phosphorylation, U937 cells displayed a significant increase in phospho-mTOR levels following Cyclopamine exposure under both oxygen conditions (Figure 9B). This opposite trend suggests a cell-type-specific uncoupling between AMPK activation and mTOR inhibition, implying that U937 cells maintain mTOR signaling despite energetic stress and AMPK activation.

Despite this unexpected mTOR behavior, Cyclopamine significantly decreased phosphorylation of the canonical mTOR downstream targets 4EBP1 (Figure 9C) and p70S6K (Figure 9D), under both normoxic and hypoxic conditions. Notably, this pattern mirrors the responses seen in CML lines, indicating that mTOR output is functionally suppressed even when phospho-mTOR levels do not decrease.

### 2.8. Smo Knock-Out Clones Confirm Metabolic Responses in K562 Cells in Hypoxic Conditions

To further corroborate the results obtained with Cyclopamine and to exclude potential non-specific toxic effects often associated with pharmacological compounds, we used stable Smo knock-out (KO) clones generated in K562 cells using a CRISPR/Cas9 approach.

**Figure 10:**
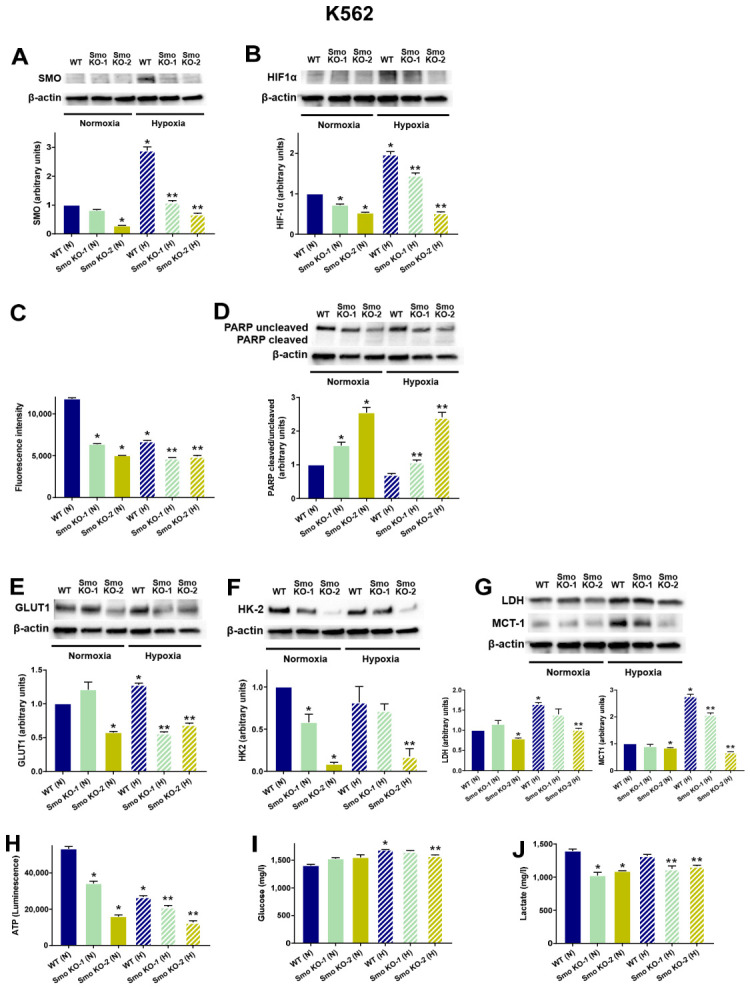
Smo suppression reveals altered metabolic responses in K562 cells, in both normoxia and hypoxia. Wild-type K562 cells and two clones of SMO CRISPR Knockout K562 Stable Cell Line were analyzed after 48 h treatment under normoxia or hypoxia. Protein expression levels of SMO (**A**), HIF-1α (**B**), PARP uncleaved and cleaved (**D**), GLUT1 (**E**), HK2 (**F**), LDH and MCT1 (**G**) were determined by Western blotting. β-actin was used as loading control. The same β-actin loading control was used for target proteins detected on membranes that were stripped and reprobed with different antibodies. Blots are representative of three independent experiments. (**C**) Cell viability was assessed using the CyQuant assay. Measurements of ATP (**H**), glucose (**I**) and lactate (**J**) levels were done by using ATP-Lite and Amplex-red glucose or lactate detection assays. Means ± SEM are presented (*n* = 3; * *p* ≤ 0.05 indicates a statistically significant difference from normoxic control, ** significantly different from hypoxic control). WT = control wild-type K562 cells, Smo KO-1 = SMO CRISPR Knockout K562 Stable Cell Line-T2-10, Smo KO-2 = SMO CRISPR Knockout K562 Stable Cell Line-T2-14, N = normoxia, H = hypoxia.

As shown in Figure 10A, Western blot analysis confirmed the efficient reduction in Smo protein levels in stable KO clones compared to control cells.

Under hypoxic conditions, stabilization of HIF-1α was observed in wild-type (WT) cells, whereas its expression was markedly reduced in Smo-deficient cells (Figure 10B), consistent with a defective hypoxia response following Hedgehog signaling loss.

Proliferation assay revealed that genetic silencing of Smo significantly impaired K562 cell growth under both oxygen conditions (Figure 10C), relating to the anti-proliferative effects of Cyclopamine. This reduction in viability was associated with increased levels of cleaved PARP (Figure 10D).

The genetic inhibition of Smo also induced profound metabolic alterations, especially in hypoxia, that mirrored the effects of chemical antagonism. We observed a decreased expression of key glycolytic markers, such as GLUT1 (Figure 10E) and HK2 (Figure 10F). Additionally, lactate metabolism was significantly affected: Smo suppression led to a decrease in the protein levels of LDH and the monocarboxylate transporter MCT1 (Figure 10G).

These changes were supported by functional assays demonstrated a significant reduction in ATP production (Figure 10H) and lactate efflux (Figure 10J) under normoxia and hypoxia. Notably, glucose consumption remained largely unaffected in Smo KO clones under normoxia, whereas a significant reduction was observed exclusively under hypoxic conditions (Figure 10I).

Collectively, these results show that the genetic inactivation of Smo closely mirrors the effects obtained through pharmacological inhibition, confirming that the Hh pathway is a critical regulator of the glycolytic shift required for adaptation to the hypoxic microenvironment.

### 2.9. Genetic Suppression of Smo Validates the Modulation of the AMPK-mTOR Axis

To further confirm the signaling mechanisms involved, we analyzed the AMPK-mTOR pathway in stable K562 Smo KO clones. The results obtained were consistent with those observed following Cyclopamine treatment.

**Figure 11:**
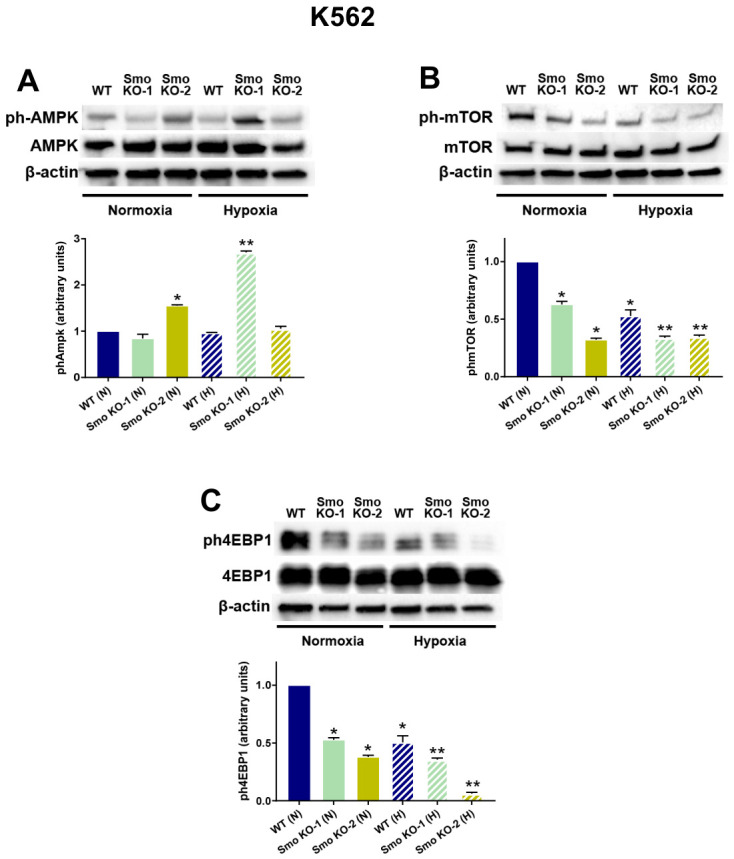
AMPK–mTOR signaling in SMO Knockout K562 Stable Cell Line. Wild-type K562 cells and two clones of SMO CRISPR Knockout K562 Stable Cell Line were analyzed in normoxic or hypoxic conditions for 48 h. Protein expression levels of ph-AMPK/AMPK (**A**), ph-mTOR/mTOR (**B**), and ph4EBP1/4EBP1 (**C**) were determined by Western blotting. β-actin was used as loading control. Blots are representative of three independent experiments. Means ± SEM are presented (*n* = 3; * *p* ≤ 0.05 indicates a statistically significant difference from normoxic control, ** significantly different from hypoxic control). WT = control wild-type K562 cells, Smo KO-1 = SMO CRISPR Knockout K562 Stable Cell Line-T2-10, Smo KO-2 = SMO CRISPR Knockout K562 Stable Cell Line-T2-14, N = normoxia, H = hypoxia.

As shown in Figure 11A, the genetic loss of Smo led to a significant increase in AMPK phosphorylation (p-AMPK) under both normoxic and hypoxic conditions.

Conversely, but consistent with the pharmacological data, genetic suppression of Smo resulted in a marked decrease in mTOR phosphorylation (Figure 11B) in both oxygen environments.

Finally, we evaluated the phosphorylation of 4EBP1, a key downstream effector of mTOR. As illustrated in Figure 11C, Smo KO clones exhibited a significant reduction in ph-4EBP1 levels, mirroring the inhibitory effects observed with Cyclopamine.

Collectively, these data provide additional evidence that the Smo-dependent Hh pathway regulates K562 cell metabolism and survival through the modulation of the AMPK-mTOR signaling axis.

## 3. Discussion

The Hh signaling pathway regulates key cellular mechanisms, including cell migration, proliferation, and survival [39]. This pathway plays a critical role in embryonic development and tissue homeostasis, but its aberrant activation has been implicated in various cancers, including chronic myeloid leukemia. Previous studies have demonstrated that Cyclopamine, a plant-derived steroidal alkaloid that inhibits Hh signaling by binding to the Smo receptor, induces apoptosis in K562 cells in a dose- and time-dependent manner [40]. We selected a Cyclopamine concentration of 20 µM based on previously published studies, including work from our laboratory, demonstrating effective inhibition of Hedgehog signaling and induction of apoptosis in cancer cell models [12,13,41,42]. Our study confirms previous observations by showing that Cyclopamine treatment was associated with reduced cell viability and changes in apoptosis- and autophagy-related markers in K562 cells [4,40], and we report this effect for the first time in KU812 cells. Similar effects were also observed in U937 cells, which are Philadelphia chromosome-negative and lack BCR-ABL1, indicating that Hedgehog pathway inhibition can affect myeloid leukemia cells independently of BCR-ABL1 status.

The bone marrow microenvironment is characterized by a gradient of oxygen tension, with concentrations ranging from 1% to 7%. This hypoxic environment plays a crucial role in the regulation of hematopoiesis and the survival of leukemic cells [43,44]. To mimic the hypoxic conditions experienced by myeloid cells in the bone marrow, we conducted our experiments at 1% O_2_ concentration. Given the complexity and heterogeneity of the bone marrow microenvironment, including the presence of dynamic oxygen gradients, more advanced experimental models may be required to fully recapitulate its physiological conditions. Nevertheless, a fixed 1% O_2_ concentration is widely accepted as a standard and reliable in vitro model for investigating cellular responses to hypoxia.

We observed a reduction in cell proliferation under hypoxic conditions compared to normoxic conditions. However, no significant increase in cell death was observed within 96 h of incubation. Our findings demonstrate that Cyclopamine reduces cell proliferation under hypoxic conditions, which is accompanied by markers of cellular stress and apoptotic signaling rather than a major autophagic response in K562, KU812, and U937 cells. The treatment with the Smoothened agonist SAG was able to reverse this effect, supporting the interpretation that the observed effects are consistent with modulation of Hh signaling. This observation suggests that Hh signaling may play a role in regulating the balance between apoptosis and autophagy in myeloid leukemia cells under hypoxic stress, regardless of Philadelphia chromosome expression.

To investigate the potential mechanisms underlying myeloid malignancy cell survival under hypoxia, we focused on metabolic reprogramming, which remains largely unexplored, especially in KU812 and U937 cells.

Cancer cells, including CML and AML cells, often undergo metabolic reprogramming, shifting their energy production towards glycolysis to support rapid proliferation and survival in challenging microenvironments. This metabolic shift, known as the Warburg effect, is characterized by increased glucose uptake and lactate production, even in the presence of oxygen. Our data identify an association between pharmacological modulation of Hh signaling and metabolic changes compatible with hypoxic adaptation in human myeloid leukemia cells. Specifically, we observed that Cyclopamine treatment significantly reduced glucose consumption, lactate production, and ATP synthesis in K562, KU812, and U937 cells, particularly under hypoxic conditions. This metabolic shift was accompanied by alterations in the expression of key glycolytic regulators. Indeed, Cyclopamine treatment significantly reduced the expression of GLUT1, HK2, LDH, and MCT1 across all three cell lines under both normoxic and hypoxic conditions. The increased reliance on glycolysis in cancer cells leads to elevated lactate production, which can acidify the tumor microenvironment. To maintain intracellular pH homeostasis, cancer cells upregulate the expression of pH-regulating enzymes, such as CAIX and CAXII. In our study, we observed a similar trend, with CAIX and CAXII expression decreasing following Cyclopamine treatment. This observation is consistent with previous reports demonstrating a link between Hh signaling and CA expression in other cancer cell types [10,11,12,13,14]. Our findings provide evidence consistent with an association between Hh signaling, hypoxia, and metabolic regulation in myeloid leukemia cell lines, highlighting a potentially conserved mechanism across different leukemia subtypes.

To further investigate the role of Hh signaling in the metabolic adaptation of CML cells to hypoxia, we stimulated the pathway using the Smo agonist SAG. Stimulation of Hh signaling with SAG produced the opposite effects compared to Cyclopamine treatment. These observations are consistent with a contribution of Hh signaling to the metabolic phenotype observed under our experimental conditions. Co-administration of SAG and Cyclopamine reversed the effects observed with Cyclopamine alone, confirming the specificity of Hh pathway modulation on the observed metabolic changes.

Recent studies have revealed a non-canonical mechanism by which Hh signaling regulates cellular metabolism. This mechanism involves a cilium-dependent axis that includes Smo, calcium ions (Ca^2+^), and AMPK, leading to rapid metabolic reprogramming similar to the Warburg effect [38]. However, the interplay between this non-canonical Hh signaling pathway and cellular metabolism in myeloid malignancies, particularly under hypoxic conditions, remains poorly understood. Our study provides the first evidence that Cyclopamine enhances AMPK phosphorylation in myeloid cells under both normoxic and hypoxic conditions. This finding suggests that Cyclopamine could simultaneously inhibit canonical Hh signaling, as evidenced by the downregulation of Smo and Gli expression, and activate non-canonical Hh signaling pathways, as proposed by Teperino et al. [38]. However, the present data do not allow us to establish whether this represents direct activation of the non-canonical pathway or an indirect consequence of Smo inhibition.

The mTOR and AMPK signaling pathways play opposing roles in regulating cellular growth and metabolism. mTOR promotes anabolic processes in nutrient-rich conditions, while AMPK activates catabolic pathways during nutrient or energy stress. In line with this, we observed that Cyclopamine reduced mTOR phosphorylation in CML cells (K562 and KU812) under both normoxic and hypoxic conditions, consistent with its role in inhibiting cell proliferation and survival [45]. This was accompanied by a reduction in the phosphorylation of downstream mTORC1 targets, including 4EBP1 and p70S6K, further supporting the inhibition of mTORC1 signaling. In contrast, in U937 cells, Cyclopamine increased phosphorylation of both mTOR and AMPK, while downstream mTORC1 substrates 4EBP1 and p70S6K were decreased. This apparent discrepancy may arise because mTOR phosphorylation does not always reflect mTORC1 functional activity. In U937 cells, AMPK could phosphorylate the mTORC1 scaffold protein Raptor, which promotes binding and inhibits mTORC1 kinase activity toward downstream targets such as 4EBP1 and p70S6K, even when mTOR itself remains phosphorylated [46]. Such a mechanism, together with potential compensatory feedback loops or altered substrate accessibility, could explain the observed pattern in U937 cells. However, as no genetic or mechanistic validation was performed in U937 cells, the molecular basis of this response remains unclear. Further studies will be required to clarify the mechanisms underlying the distinct response observed in U937 cells. Nevertheless, these findings indicate cell-type-dependent regulation of the AMPK–mTOR axis, with Cyclopamine ultimately suppressing mTORC1-dependent translation machinery through possibly distinct mechanisms in CML and non-CML myeloid cells.

These observations raise the possibility that, in addition to the canonical Smo–Gli pathway, the AMPK–mTOR pathway may contribute to the cellular responses associated with Smo inhibition under hypoxic conditions. Although the present data do not establish a causal role for this pathway, they support further investigation of AMPK–mTOR signaling as a potential therapeutic target for modulating metabolic adaptation in myeloid malignancies within the hypoxic bone marrow microenvironment.

A schematic summary of the proposed Hedgehog–AMPK–mTOR relationship in CML cells is provided in Supplementary Figure S2. Nevertheless, it is important to note that our data remain largely correlative; since no pharmacological or genetic rescue experiments targeting AMPK or mTOR were performed, we cannot formally establish a direct causal hierarchy or prove that this axis strictly mediates the metabolic effects of Smo inhibition.

Importantly, in K562 cells, the main findings related to metabolic regulation and AMPK–mTOR axis modulation were further confirmed using CRISPR/Cas9-mediated Smo knockout models. These genetic data increase confidence that the metabolic changes observed under hypoxia are related, at least in part, to Smo inhibition rather than to off-target effects of Cyclopamine. Notably, this genetic validation was performed only in K562 cells, whereas the findings obtained in KU812 and U937 cells are based on pharmacological inhibition with Cyclopamine and should therefore be interpreted with appropriate caution.

In conclusion, our in vitro study provides evidence of an association between Hedgehog pathway modulation, metabolic alterations and changes in AMPK-mTOR-related signaling under hypoxic conditions in myeloid leukemia cell models. These findings support the concept that the hypoxic bone marrow microenvironment may influence leukemia cell behavior through metabolic adaptation and are consistent with the hypothesis that Hedgehog signaling may contribute to this process. Further studies using primary patient samples and in vivo models will be required to determine the clinical relevance of these observations.

## 4. Materials and Methods

### 4.1. Cell Culture Conditions and Treatments

K-562 and KU-812 cell lines were purchased from Leibniz Institute DSMZ, Braunschweig, Germany, and U937 cell line from ATCC (ATCC^®^ CRL-1593.2). Cells were cultured in RPMI 1640 supplemented with Fetal Bovine Serum (FBS) (10% for K-562 and U937 and 20% for KU-812), penicillin (100 U/mL)/streptomycin (100 µg/mL) and 2 mM L-Glutamine (all purchased from Euroclone, Devon, UK).

Cells were incubated at 37 °C under either normoxia (21% O_2_), corresponding to a pO_2_ of ~140 mmHg, or hypoxia (1% O_2_), corresponding to a pO_2_~7 mmHg, by using a hypoxic chamber (Coy Laboratory Products, Grass Lake, MI, USA).

Where indicated, cells were treated either with Cyclopamine hydrate (Cyclo) (Sigma-Aldrich, St. Louis, MO, USA), which blocks the Hh signaling by directly binding SMO, at a final concentration of 20 µM, or with a SMO agonist, Sag dihydrochloride (SAG), used at a final concentration of 300 nM, or with the combination of both Cyclo and SAG. Chemical compounds were purchased from Sigma-Aldrich, St. Louis, MO, USA.

### 4.2. Cell Viability and Proliferation Assays

Cells were seeded in 96-well plates incubated for 8, 24, 48, 72 and 96 h under either normoxic or hypoxic conditions. Then, cell viability was analyzed by Trypan Blue exclusion assay, as previously described [47]. Briefly, a Bio-Rad TC20™ automated cell counter (Biorad laboratories, Bio-Rad, Hercules, CA, USA) provided viable cell count by excluding dead cells labeled with Trypan Blue and using a digital image analysis algorithm. Cell viability was expressed as percentage of live cells.

In addition, after 48 h of exposure to either normoxia or hypoxia, in the presence or not of Cyclo, SAG, or both chemical compounds, cell proliferation was analyzed by using the CyQUANT™ Direct Cell Proliferation Assay (Thermo Fisher Scientific, Cleveland, OH, USA), according to the manufacturer’s instructions. This assay provides staining of nucleic acids with a live cell-permeable fluorescent dye. Fluorescence intensity was measured with a fluorescence microplate reader (FluostarOptima, BMG LABTECH, Durham, NC, USA) and cell viability was expressed as fluorescence intensity.

### 4.3. Western Blotting

To perform Western blot analysis, cells, treated or untreated with Cyclo, SAG, or both chemical compounds, were exposed under either normoxic or hypoxic conditions for 48 h. Then, cells were promptly lysed in RIPA buffer (Cell signaling, Denver, CO, USA) supplemented with a cocktail of protease inhibitors (Sigma-Aldrich, St. Louis, MO, USA).

For each sample, equal amounts of total protein, determined with Micro BCA Protein Assay Reagent kit (Rockford, IL, USA), were loaded onto SDS-PAGE gel, and at the end of the electrophoretic run, proteins were transferred onto 0.2 µm nitrocellulose membranes (Bio-Rad Laboratories, Hercules, CA, USA). Membranes were incubated with the following primary antibodies: HIF-1α (BD Biosciences, San Jose, CA, USA); SMO (Thermo Fisher Scientific, Waltham, MA, USA); GLI-1, BNIP3, PARP, GLUT1, HK-2, LDHA, CAIX, CAXII, phospho-AMPKα, AMPKα, phospho-mTOR, mTOR, phosphor-4EBP1, 4EBP1, phospho-p70S6K, p70S6K (all purchased from Cell signaling, Denver, CO, USA); MCT-1 (Sigma-Aldrich, St. Louis, MO, USA) and β-actin (Sigma-Aldrich, St. Louis, MO, USA). β-actin was used as loading control. When multiple proteins with distinct molecular weights were analyzed on the same membrane, membranes were stripped and reprobed with different antibodies, and the same β-actin loading control was used for the corresponding targets. Anti-mouse and anti-rabbit IgG-HRP (Cell signaling, Denver, CO, USA) were used as secondary antibodies. Image acquisition was performed by using ChemiDoc™ MP System and blots were quantified by using Image Lab software (version 6.1.0 build 7, Bio-Rad Laboratories, Hercules, CA, USA).

### 4.4. ATP Detection Assay

Luminescence ATP detection assay was performed to determine ATP concentrations in cell lines treated or untreated with Cyclo, SAG, or both chemical compounds. To this end, an ATPlite assay kit was purchased from PerkinElmer (Shelton, CT, USA) and cells, treated as previously described, were seeded onto black 96-well plates. After 48 h incubation under either normoxic or hypoxic conditions, the assay was executed according to the manufacturer’s instructions. Luminescence was measured with a microplate reader (FluostarOptima, BMG LABTECH, Durham, NC, USA) and ATP levels were expressed as luminescence produced. ATP levels were not normalized to the cell number measured after the 48-h incubation, since cell proliferation during the experimental period results in changes in cell number over time, making the endpoint cell count unsuitable for normalization.

### 4.5. Immunofluorescence

Immunofluorescence slides (Epredia Netherlands B.V., Breda, The Netherlands) were pre-coated with Poly-D-Lysine (Sigma Aldrich, Saint Louis, MO, USA) for 20 min at room temperature in order to facilitate cell adhesion. After 48 h of treatment, cells were harvested and seeded onto the slides allowing them to attach for 20 min at room temperature. Fixation and permeabilization with methanol at −20 °C were performed. GLUT1, HK-2, LDHA (Cell signaling, Denver, CO, USA) and MCT-1 (Sigma-Aldrich, St. Louis, MO, USA) were used as primary antibodies (diluted 1:300 in PBS 2% BSA). After overnight incubation at 4 °C in a humidified chamber and 2 washing steps, secondary antibodies (antimouse 488 conjugated or antirabbit 550 conjugated, both purchased from Thermo Fisher Scientific, Cleveland, OH, USA) were added and slides were incubated at room temperature for 1 h. Nuclei were stained with Hoechst 33342 (Fluka, Sigma Aldrich, Saint Louis, MO, USA) 1 mg/mL (1:1000) for 4 min at room temperature. After final washing, cover slides were mounted with Mowiol^®^ 4-88 Reagent (EMD Millipore, Burlington, MA, USA). Images with 60× magnification were acquired with an Olympus IX81 microscope (Olympus, Tokyo, Japan) and analyzed with ImageJ software 1.53K (http://imagej.nih.gov/ij/docs/index.html) (accessed on 5 February 2024). Fluorescence intensity was expressed by corrected total cell fluorescence (CTCF) = integrated density − (area of selected cell × mean fluorescence of background readings).

### 4.6. Amplex-Red Glucose and Lactate Detection Assays

Glucose consumption and lactate production were analyzed by performing Amplex-red assays, as previously reported [48], in all cell lines, in the presence or not of Cyclo, SAG or both chemical compounds. Amplex-red assays couple the oxidation of glucose or lactate, by glucose oxidase and lactate oxidase respectively, which results in oxygen peroxide generation. H_2_O_2_ in a reaction catalyzed by peroxidase (HRP) converted Amplex-red reagent into fluorescent Resorufin. To this end, Ampliflu-Red, HRP type VI, glucose oxidase from *Aspergillus niger*, lactate oxidase from *Aerococcus viridans* and calcium-lactate were purchased from Sigma-Aldrich, St. Louis, MO, USA.

Briefly, cells were incubated for 48 h under either normoxia or hypoxia. Then, supernatants from each sample were diluted in a 0.1 M sodium phosphate buffer (pH 7.4) and transferred into a black 96-well plate. A working solution of 100 µM Amplifu-Red reagent, 0.2 U/mL HRP and 2 U/mL glucose or lactate oxidase (for glucose and lactate detection respectively) was added to each well and the plate was incubated for 30 min at room temperature, protected from light.

Positive (H_2_O_2_) and negative (sodium phosphate buffer) controls were used. Standard curves were obtained by preparing 4 series of 2-fold serial dilutions (starting from 100 µM for the glucose and from 10 µM for the lactate) in sodium phosphate buffer.

Fluorescence (λex: 530 nm and λem: 590 nm) was measured at multiple time points with a fluorescence microplate reader (FluostarOptima, BMG LABTECH, Durham, NC, USA). Glucose consumption and lactate production were expressed as mg/L. Glucose consumption and lactate production were not normalized to the cell number at the end of the 48-h incubation, as cell number changes throughout the observation period and therefore does not accurately reflect the number of metabolically active cells over the entire assay duration.

### 4.7. Generation of Smo KO K-562 Cell Line

The SMO CRISPR knockout K562 stable cell line (Applied Biological Materials Inc., Cat. No. C208) was generated via lentiviral transduction of K562 cells targeting the SMO gene (Accession No. NM_005631.5). Lentiviral packaging was performed using a third-generation system according to the manufacturer’s protocols using the T2 sgRNA sequence 5′-GGGTTGTCTGTCCGAACCAA-3′. Following transduction and selection, two specific clones (T2-10 and T2-14) were isolated and characterized as harboring biallelic compound heterozygous frameshift mutations. Genomic DNA was validated via PCR and Sanger sequencing using the T2 forward primer 5′-AGTGTATCTCCAGCCACCCTGCCATGCTACCTAG-3′ and reverse primer 5′-GGCCATTCTGTGAGGTTAGGGTTATTCTGCTGACA-3′. The resulting clones were maintained in RPMI 1640 medium supplemented with 10% fetal bovine serum, 1% penicillin/streptomycin, and 0.4 µg/mL puromycin at 37.0 °C in a humidified atmosphere with 5% CO_2_.

### 4.8. Statistical Analysis

Data are presented as the mean ± SEM of at least three independent experiments. Pairwise comparisons were evaluated using Student’s *t*-test, and *p*-values were subsequently adjusted for multiple comparisons using the False Discovery Rate (FDR) correction. All statistical analyses were performed with GraphPad Prism (Version 9.5.1 (733), San Diego, CA, USA) and R version 4.3.1. An adjusted *p*-value ≤ 0.05 was considered statistically significant.

## Data Availability

The data generated during the current study are available from the corresponding author on reasonable request.

## Supplementary Materials

The following supporting information can be downloaded at https://www.mdpi.com/article/10.3390/ijms27146324/s1.

## Abbreviations

The following abbreviations are used in this manuscript:

ALL	acute lymphoid leukemia
AML	acute myeloid leukemia
AMPK	AMP-activated protein kinase
BNIP3	Bcl-2/adenovirus E1B 19 kDa-interacting protein 3
CAs	carbonic anhydrases
CLL	chronic lymphoid leukemia
CML	chronic myeloid leukemia
FBS	Fetal Bovine Serum
GLI	glioma-associated oncogene
GLUT1	glucose transporter 1
HIF	Hypoxia-Inducible Factor
Hh	Hedgehog
HK2	hexokinase 2
HREs	hypoxia-responsive elements
HSCs	hematopoietic stem cells
LDH	lactate dehydrogenase
MCL	mantle cell lymphoma
MCT1	monocarboxylate transporter 1
MM	multiple myeloma
mTOR	mammalian target of rapamycin
PARP	Poly(ADP-ribose) polymerase
PI3K	phosphatidylinositol 3-kinase
