# Supplementary material for: Hedgehog Signaling Regulates Hypoxia-Associated Metabolic Adaptation in Myeloid Leukemia In Vitro Cell Models
Source: Int J Mol Sci. 2026 Jul 16;27(14):6324. doi: 10.3390/ijms27146324 (PMC13410166; doi:10.3390/ijms27146324)
Supplement: Supplementary file 1 [file ijms-27-06324-s001.zip › ijms-4358910-supplementary.pdf]

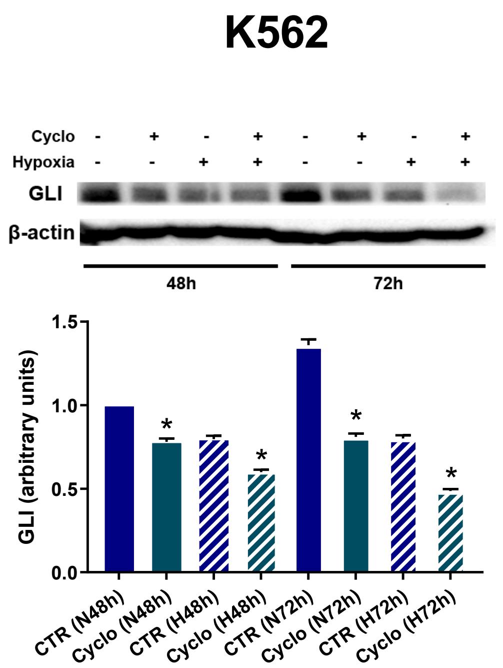

Figure S1. Cyclopamine impairs GLI expression in K562 cells under normoxic and hypoxic conditions. K562 cells were treated with Cyclopamine under normoxic or hypoxic conditions for 48 or 72 hours. Protein expression levels of GLI was analyzed by Western blotting. β-actin served as the loading control. A representative blot is shown from three independent experiments. Data represent means ± SEM (n = 3; *p ≤ 0.05 indicates statistical significance compared to control). CTR = control, Cyclo = Cyclopamine.

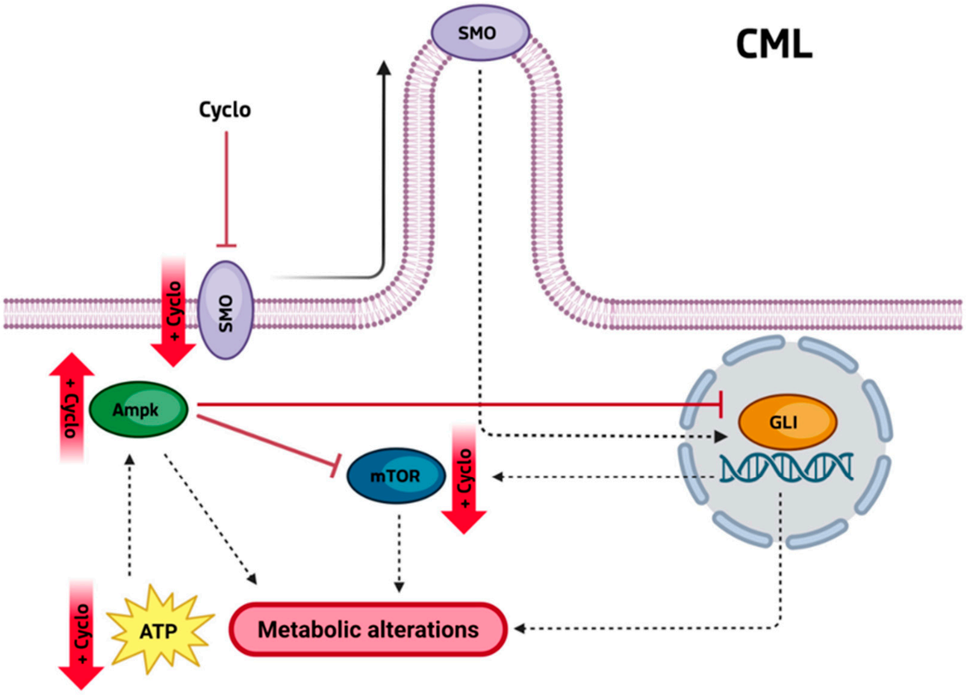

Figure S2. Schematic summary of the proposed Hedgehog–AMPK–mTOR relationship in CML cells.
